# Psychological Distancing Usage Uniquely Predicts Reduced Perceived Stress During the COVID-19 Pandemic

**DOI:** 10.3389/fpsyg.2022.838507

**Published:** 2022-02-16

**Authors:** Eva E. Dicker, Jenna S. Jones, Bryan T. Denny

**Affiliations:** Department of Psychological Sciences, Rice University, Houston, TX, United States

**Keywords:** COVID-19, emotion regulation, cognitive reappraisal, psychological distancing, stress

## Abstract

Social distancing during the COVID-19 pandemic has presented millions of people with extraordinary challenges that are associated with significant amounts of stress. Emotion regulation is crucial during this crisis as people seek to mitigate the stress and uncertainty of the present moment. In this study, we surveyed a nationally representative sample of 297 adults from the United States on their levels of perceived stress related to the COVID-19 pandemic as well as their level of engagement of different emotion regulation strategies during the pandemic. We performed multiple linear regression analyses to assess which regulation strategies were associated with individual differences in perceived stress. Among all emotion regulation strategies, psychological distancing, which involves thinking about stressful circumstances in an objective, impartial way, was uniquely associated with reductions in perceived stress due to COVID-19 across individuals. This effect was not moderated by age, gender, socioeconomic status, race/ethnicity, or trait-related difficulty in regulating emotion. Conversely, situation modification was associated with significantly greater perceived stress overall. These results suggest the broad applicability and utility of psychological distancing during pandemic-related social distancing as part of an adaptive emotion regulation toolkit and motivate the investigation of interventions involving psychological distancing in this context.

## Introduction

The ongoing coronavirus (COVID-19) pandemic has taken an extraordinary toll on individuals across the globe. Similar to previous global outbreaks (e.g., Ebola, H1N1, avian flu, and SARS), the COVID-19 pandemic has been shown to predict psychological stress, depression and anxiety in addition to substantial morbidity and mortality (Bults et al., 2011; Huang and Zhao, 2020; Jungmann and Witthöft, 2020; Li et al., 2020; Rajkumar, 2020). Restriction of regular daily activities such as school, work, and travel have been shown to be effective in preventing exposure and containing the spread of COVID-19, but these polices, collectively known as “social distancing,” are likely to contribute to the substantial perceived stress associated with the pandemic.

Indeed, since the beginning of the COVID-19 pandemic in December 2019, psychological and physiological impacts such as generalized anxiety, depression, poor health behaviors, and insomnia have been observed across the globe (Sabel et al., 2018; Alkhamees et al., 2020; Clemente-Suárez et al., 2020; Qi et al., 2020; Rossi et al., 2020; Goularte et al., 2021; Liu et al., 2021a,b; Rossi et al., 2021). A recent review of the impacts of quarantining during COVID-19 has identified several influential stressors contributing to negative psychological outcomes during pandemics (Brooks et al., 2020; Clemente-Suárez et al., 2020). Isolation limits the social resources required for coping and predicts negative mental and cardiovascular outcomes (Bavel et al., 2020; Xiao et al., 2020). In addition, the current COVID-19 pandemic has resulted in mass layoffs, pay cuts, reduced work hours and work-from-home orders (Brooks et al., 2020; Restubog et al., 2020). Indeed, both individual financial and labor market concerns are associated with negative psychological outcomes (Restubog et al., 2020), contributing to the overall stress that many have experienced during the pandemic.

The present pandemic highlights the essential role of emotion regulation as a means to help mitigate stress people are experiencing during COVID-19. Emotion regulation can be implemented via one or more regulation strategies (Gross, 1998). James Gross’ process model of emotion regulation is a useful theoretical framework through which to examine separable classes of emotion regulation strategies that individuals may implement to change how they feel (Gross, 1998, 2015). Strategies of emotion regulation described by the process model are situation selection (e.g., avoiding an emotional situation entirely), situation modification (e.g., altering the external environment), attention redeployment (e.g., shifting internal or external attention), cognitive change (e.g., cognitive reappraisal; changing the meaning of an emotional situation), and response modulation (e.g., expressive suppression) (Gross, 1998).

Broadly, prior work has shown that antecedent-focused strategies like cognitive reappraisal are often more adaptive than response-focused strategies, such as expressive suppression (a type of response modulation) (Gross and John, 2003; John and Gross, 2004). Cognitive reappraisal has been associated with reduced negative affect as well as enhanced physical and psychological health, whereas expressive suppression has been shown to be associated with poorer health outcomes (Gross and John, 2003; John and Gross, 2004).

In addition, reappraisal itself may be further characterized and operationalized as an emotion regulation strategy as a function of multiple reappraisal tactics (McRae et al., 2012), particularly including psychological distancing and reinterpretation (Denny and Ochsner, 2014). Psychological distancing involves taking on the perspective of an impartial and objective observer and/or increasing perceived physical or temporal distance from a stress inducing stimulus or situation (Denny and Ochsner, 2014; Powers and LaBar, 2019). Alternatively, reinterpretation involves reframing the situation by imagining that the situation is not as bad as it first seemed or that the situation will soon get better (Denny and Ochsner, 2014). Previous work in healthy adults has shown that, in contrast to reinterpretation training, longitudinal training in psychological distancing uniquely predicts drops in perceived stress over time, which may result from the relative stimulus independence and cognitive automatability of distancing (Denny and Ochsner, 2014). Thus, psychological distancing represents a promising emotion regulation strategy to probe in the context of stress related to COVID-19.

Previous research on the H1N1 pandemic showed how individual differences in adaptive behaviors were predicted by a variety of background factors like age, gender, socioeconomic status, and by personality factors like impulsivity and sensation seeking (Gaygisiz et al., 2012). Taha et al. (2014) further observed that emotion dysregulation was positively associated with anxiety during the H1N1 pandemic. Thus, in the current study we were interested to model trait-level individual differences in emotion dysregulation as well as person factors like age, gender, race/ethnicity, and socioeconomic status.

In order to examine associations between engagement of particular emotion regulation strategies and stress specifically related to COVID-19, in the present study we recruited a nationally representative sample of 297 individuals in the United States in May 2020 to complete a set of questionnaires assessing demographic information, trait-related emotion dysregulation; overall pandemic-related stress; and self-reports of the extent to which individuals were using a range of emotion regulation strategies during COVID-19. We predicted that engagement of reappraisal, particularly via psychological distancing, would be associated with lower pandemic-related stress, whereas other emotion regulation strategies were expected to be either unrelated or positively related to pandemic-related stress.

## Materials and Methods

### Participants

We conducted an online study via a web-based recruitment platform (i.e., Prolific)^1^ designed to recruit US nationally representative survey samples with diverse, trusted participants. Participants were required to be at least 18 years of age, be fluent in English, and provide informed consent to participate. Participants who did not meet these criteria were excluded from participation. The projected sample size was calculated via a power analysis for a small-to-medium correlation effect size (ρ = 0.2); at this effect size, 90% power (α = 0.05) to detect an effect, two-tailed, would be achieved with 258 participants. Thus, we aimed to recruit approximately 300 participants. We recruited 298 participants. One participant was excluded from analysis due to missing emotion regulation strategy usage data, resulting in a total analyzed sample of 297 participants. All participants provided informed consent in accordance with the Rice University Institutional Review Board. Participants ranged in age from 19 to 82, with a mean age of 45.32 (*SD* = 16.19). Gender, race, and ethnicity information for the sample is shown in Table 1.

**TABLE 1:** Descriptive statistics and sample characteristics.

Variable	Mean ± *SD*
Overall COVID-19 stress	52.11 ± 28.36
Situation selection	2.36 ± 2.02
Situation modification	3.91 ± 1.91
Distraction	4.84 ± 1.68
Reinterpretation	4.29 ± 1.73
Distancing	4.14 ± 1.66
Expressive suppression	3.69 ± 1.78
DERS	2.07 ± 0.66
Age	45.32 ± 16.19
Gender	
Male	147 (49.5%)
Female	145 (48.8%)
Other	5 (1.7%)
SES	5.60 ± 1.83
Race	
American Indian or Alaskan Native	2 (< 1.0%)
Asian	22 (7.4%)
Black or African American	45 (15.2%)
White-Caucasian	208 (70.0%)
More than one race	12 (4.0%)
Other	8 (2.7%)
Ethnicity	
Non-Hispanic	270 (90.9%)
Hispanic	22 (7.4%)
Decline to state	5 (1.7%)

### Self-Report Measures

In addition to demographic information, participants reported their perceived socioeconomic status (SES) via the MacArthur Scale of Subjective Social Status (Adler et al., 2000). Participants also completed a widely used trait-related measure of emotion dysregulation, the Difficulties in Emotion Regulation Scale, Short Form (DERS-SF; Gratz and Roemer, 2004; Kaufman et al., 2016). While not the focus of the current analyses, participants also completed questionnaires assessing perceived stress (Cohen et al., 1983), positive and negative affect (PANAS-SF; Watson et al., 1988), state and trait-related anxiety (STAI-6; Marteau and Bekker, 1992), depressive symptoms (CES-D; Radloff, 1977), overall reappraisal and suppression usage (ERQ; Gross and John, 2003), and quality of life (SF-36; Ware and Sherbourne, 1992).

We next assessed the primary dependent variable, overall perceived stress related to COVID-19. Specifically, we asked participants, “How much stress are you feeling overall right now due to COVID-19 and its impacts and consequences?” Participants responded using a continuous sliding scale ranging from 0 (no stress at all) to 100 (extreme stress). This questionnaire can be found in Supplementary Figure 1.

In addition to this summary measure of primary interest as a dependent variable (i.e., overall COVID-19-related stress), we next also assessed stress related to COVID-19 in specific domains (i.e., stress due to social isolation, uncertainty on the duration of the pandemic, concern for others’ wellbeing, personal health concern, and financial hardship concern) using a continuous 0–100 scale. However, these subscales for COVID-19-related stress were substantially intercorrelated with the overall COVID-19-related stress measure (Cronbach’s alpha = 0.88). Thus, the overall COVID-19-related stress measure represented the dependent variable of interest for all subsequent analyses.

Finally, we assessed the frequency with which participants engaged in a range of emotion regulation strategies derived from the Gross process model of emotion regulation (Gross, 1998). Specifically, we asked, “When you have felt stressed due to social distancing/COVID-19, how much have you:,” followed by assessment of frequency of situation selection (i.e., “changed your situation fundamentally, e.g., by traveling from a more affected area to a less affected area to reduce your risk of infection”), situation modification (i.e., “altered your situation to become less stressful/more normal, e.g., by engaging in virtual social interaction with friends and family via Zoom, FaceTime, etc. to try to make things feel more normal”), attention redeployment/distraction (i.e., “distracted yourself from crisis-related events and information and focused your attention elsewhere, e.g., by engaging in hobbies and/or consuming non-crisis related social media and entertainment”), reappraisal-by-reinterpretation (i.e., “changed how you think about the crisis itself in ways that help you feel less negative or more positive by reframing the situation in your mind, e.g., by thinking about how social distancing practices are beneficial to you and your community; contemplating how much money you may be saving on gas or other things; or thinking about how we’ll pull together and get through this eventually”), reappraisal by-distancing (i.e., “changed how you think about the crisis itself by trying to objectively and calmly process what’s going on without judgment, e.g., by changing the way you think from a first-person, immersed perspective to a more objective, third-person perspective on the crisis”), and response modulation/expressive suppression (i.e., “tried to directly modify your emotional responses to the crisis, e.g., by trying not to show any outward signs of negative emotion and keeping everything inside”). Responses for each question were made on a 7-point Likert scale, ranging from 1 (not at all) to 7 (very much so). Finally, we included a free-response option to assess if participants had tried any other strategies to reduce stress during COVID-19. This questionnaire can be found in Supplementary Figure 2.

### Statistical Analysis

Two participants showed elevated DERS scores (≥ 3 standard deviations from the mean). Therefore, in order to not exclude these participants and to include all analyzable 297 participants in the analysis, we performed robust regression using M-M estimation via lmrob (Maechler et al., 2020) in R Studio. *A priori* predictors were frequency of usage of the 6 emotion regulation strategies described above (i.e., situation selection, situation modification, distraction, reinterpretation, distancing, and expressive suppression). *A priori* covariates were age, gender (dichotomized as female vs. non-female given a low number of participants indicating other gender/decline to state), community-level SES (Adler et al., 2000), race/ethnicity (dichotomized as white, non-Hispanic vs. non-white, non-Hispanic given sample limitations) and trait-related difficulties in regulating emotion (Gratz and Roemer, 2004; Kaufman et al., 2016). The dependent variable was overall COVID-19-related stress. All analyses were conducted using R Studio Version 1.3.1093 (RStudio Team, 2020).

## Results

Descriptive statistics for each measure were calculated (Table 1), and raw data are registered at the Open Science Framework^2^. We first examined zero-order correlations among all variables (see Supplementary Table 1). Notably, frequency of situation selection, situation modification, distraction, and expressive suppression as well as extent of difficulty regulating emotion were significantly positively correlated with overall COVID-19-related stress (all *p* < 0.05, two-tailed); no other variables showed significant zero-order correlations with overall COVID-19-related stress in either direction (see Supplementary Table 1).

We next examined our *a priori* robust multiple regression model incorporating standardized regressors for all six emotion regulation strategy use frequencies as well as age, gender, community-level SES, race/ethnicity, and trait-related difficulty regulating emotion in predicting overall COVID-19-related stress. This model met all assumptions for multiple regression. The variance inflation factor for each predictor was low (all < 2.0), suggesting low multicollinearity. The data met the assumption of independent errors (Durbin-Watson value = 1.96, *p* = 0.36).

Regression results for our *a priori* model (*R*^2^ = 0.25; Adjusted *R*^2^ = 0.22) are shown in Table 2. Greater use of situation modification was associated with greater overall COVID-19-related stress (β = 0.20, *p* < 0.01, 95% CI [0.06, 0.33]). Further, as expected, increased trait-related difficulty in regulating emotion was also significantly associated with greater overall COVID-19-related stress (β = 0.44, *p* < 0.001, 95% CI [0.31, 0.57]). Conversely, greater use of distancing was unique among all emotion regulation strategies, and unique among all regression variables overall, in significantly predicting lower overall COVID-19-related stress (β = -0.14, *p* < 0.05, 95% CI [-0.28, 0.00]).

**TABLE 2:** Robust multiple regression results.

Predictors	B	Std error	β	Lower 95% CI	Upper 95% CI	*t*	*p*
(Intercept)	–13.89	0.09	0.06	–0.11	0.23	0.74	0.46
Situation selection	0.71	0.05	0.05	–0.05	0.15	0.96	0.34
Situation modification	2.90	0.07	0.20**	0.06	0.33	2.90	0.00
Distraction	1.75	0.07	0.10	–0.03	0.24	1.53	0.13
Reinterpretation	1.14	0.08	0.07	–0.08	0.22	0.89	0.38
Distancing	–2.39	0.07	−0.14*	–0.28	0.00	–2.04	0.04
Expressive suppression	0.96	0.07	0.06	–0.08	0.20	0.84	0.40
DERS	18.83	0.07	0.44***	0.31	0.57	6.53	0.00
Age	0.10	0.07	0.06	–0.07	0.19	0.87	0.38
Gender	–3.72	0.11	–0.13	–0.35	0.09	–1.19	0.23
SES	0.76	0.06	0.05	–0.07	0.17	0.81	0.42
Race/ethnicity	0.05	0.13	0.00	**−**0.24	0.25	0.01	0.99

*p < 0.05, **p < 0.01, ***p < 0.001.

Given that usage of distancing was unique in predicting lower overall COVID-19-related stress, we were motivated to examine an additional multiple regression model incorporating all of the regressors from the *a priori* model as well as separate interaction terms between distancing usage and each covariate (i.e., age, gender, community-level SES, race/ethnicity, and trait-related difficulty regulating emotion) in order to assess whether any of these demographic variables may moderate the relationship between distancing and overall COVID-19-related stress. This model met all assumptions for regression. Regression results for this model are shown in Supplementary Table 2. No interactions between distancing usage and any of the covariates above were significant (all *p* > 0.26; Supplementary Table 2).

## Discussion

This study investigated associations between individuals’ usage of a range of emotion regulation strategies and overall stress experienced during the COVID-19 pandemic. In addition to the threat of infection and illness, the toll of quarantine policies have created acute psychological consequences across the globe during the COVID-19 pandemic (Balsamo and Carlucci, 2020). The present results showed that greater usage of psychological distancing uniquely predicted lower levels of overall COVID-19-related stress. Conversely, greater usage of situation modification was significantly associated with higher levels of pandemic-related stress; while the causality of this result is unclear, it may reflect the dissonance of trying to make things feel more normal with virtual means (e.g., Zoom calls standing in as limited replacements for prior social interactions).

The unique effect of psychological distancing in mitigating COVID-19-related stress was not moderated by age, gender, socioeconomic status, race/ethnicity, or overall difficulties regulating emotion in general, suggesting fairly broad generalizability. The present results using a United States sample are also consistent with recent work examining the relationship between psychological distancing and anxiety in Chinese participants in the context of COVID-19 (Zheng et al., 2020). Zheng and colleagues reported that psychological distancing mediated the relationship between COVID-19 severity and anxiety, with greater engagement of psychological distancing associated with diminished anxiety. Thus, it’s possible that the present results may be cross-cultural as well, although future work should examine these relationships in additional cultural contexts (Carlucci et al., 2020). The present findings are also consistent with those of Jungmann and Witthöft (2020), which reported that broadly defined adaptive emotion regulation (including reappraisal, among others) buffered pandemic-related anxiety during COVID-19. The present results further characterize this relationship by suggesting a unique, adaptive role for distancing in particular.

Indeed, even among reappraisal tactics (i.e., distancing and reinterpretation), distancing usage was shown to uniquely predict lower COVID-19-related stress. Reinterpretation usage was not significantly predictive of pandemic-related stress. This aligns with previous research that has identified distancing as a frequently adaptive strategy for high stress situations overall (Denny and Ochsner, 2014; Kross and Ayduk, 2017) as well as a relatively adaptive strategy for high stress situations in comparison to reinterpretation (Denny and Ochsner, 2014). As distancing is thought to represent one of the key “active ingredients” driving the benefits of mindfulness practices (Kross and Ayduk, 2017), the present results suggest that distancing may be of particular relevance when used to cope with the significant stress associated with the COVID-19 pandemic.

### Limitations and Future Directions

This present study has some limitations. It’s important to note that the present results are correlational and cross-sectional. While it’s plausible that emotion regulation usage may have causally impacted pandemic-related stress, such causality cannot be inferred from the present results. We also used an exploratory single item measure of COVID-19 related stress, and our emotion regulation scale was designed to assess frequency of strategy use, not degree of success in usage. Future studies may probe usage success as well as usage frequency. In addition, future studies should utilize larger sample sizes that allow for more specific modeling of gender as well as racial and ethnic differences.

While correlational, the present results identify psychological distancing as a plausible causal factor and candidate psychological mechanism through which individuals regulated their COVID-related stress. These results may motivate future longitudinal, experimental work to determine if receiving training in psychological distancing may be causally related to changes in pandemic-related stress.

## Conclusion

The present study found that greater usage of psychological distancing was uniquely associated with lower overall stress related to the COVID-19 pandemic, whereas situation modification was related to higher overall COVID-19-related stress. These findings implicate psychological distancing as a promising candidate to examine as a target in future emotion regulation training work focused on the impact of stressful situations like the COVID-19 pandemic. While the present pandemic has been profoundly challenging for individuals and society in so many ways, increased knowledge of potentially adaptive regulatory options may help people prepare to cope with current and future stressful circumstances.

## Data Availability Statement

The datasets presented in this study can be found in online repositories. The name of the repository and accession number can be found in the article.

## Ethics Statement

The studies involving human participants were reviewed and approved by the Rice University Research Compliance. The patients/participants provided their written informed consent to participate in this study.

## Author Contributions

JJ conducted the data collection. ED conducted the data analysis. ED and JJ wrote the manuscript under the supervision of BD. All authors contributed to design of the study, reviewed, and approved of the final manuscript.

## Conflict of Interest

The authors declare that the research was conducted in the absence of any commercial or financial relationships that could be construed as a potential conflict of interest.

## Publisher’s Note

All claims expressed in this article are solely those of the authors and do not necessarily represent those of their affiliated organizations, or those of the publisher, the editors and the reviewers. Any product that may be evaluated in this article, or claim that may be made by its manufacturer, is not guaranteed or endorsed by the publisher.

## Footnotes

1
https://www.prolific.co

2
https://osf.io/tg86c/?view_only=74f5a410dfdb4652852b12caf95edc41
