# Supplementary material for: Psychological Distancing Usage Uniquely Predicts Reduced Perceived Stress During the COVID-19 Pandemic
Source: Front Psychol. 2022 Feb 16;13:838507. doi: 10.3389/fpsyg.2022.838507 (PMC8888423; doi:10.3389/fpsyg.2022.838507)
Supplement: Supplementary file 1 [file Table_1.docx]

Supplementary Material

Supplemental Figure 1. Overall COVID-19 related stress questionnaire.


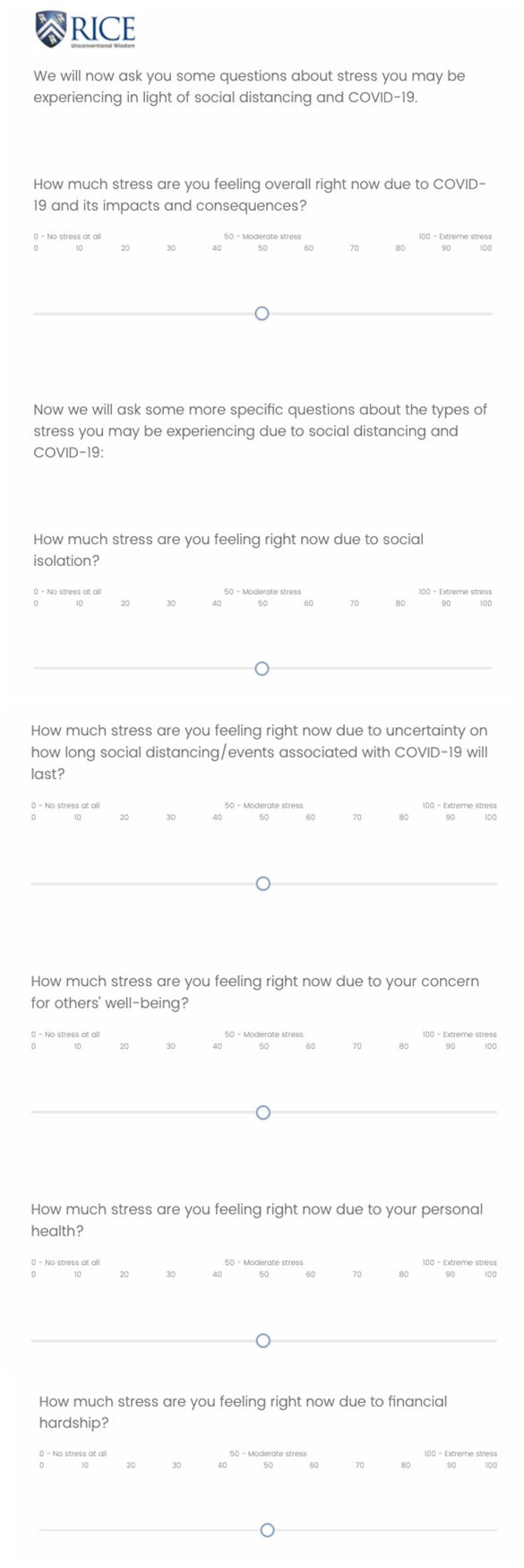


Supplemental Figure 2. Emotion regulation frequency questionnaire.


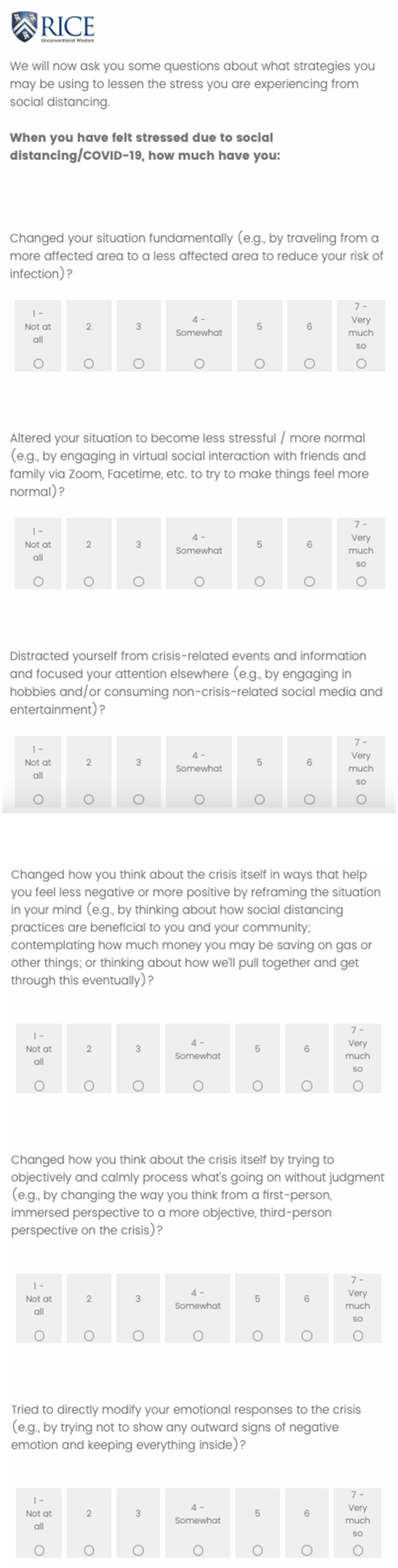


Supplemental Table 1. Zero-order correlations between all variables.

| **Measure** | **1** | **2** | **3** | **4** | **5** | **6** | **7** | **8** | **9** | **10** |
| --- | --- | --- | --- | --- | --- | --- | --- | --- | --- | --- |
| 1. Overall COVID-19 related stress | — |  |  |  |  |  |  |  |  |  |
| 2. Situation selection | 0.12 * | — |  |  |  |  |  |  |  |  |
| 3. Situation Modification | 0.19 ** | 0.25 *** | — |  |  |  |  |  |  |  |
| 4. Distraction | 0.18 ** | 0.14 * | 0.35 *** | — |  |  |  |  |  |  |
| 5. Reinterpretation | 0.07 | 0.20 *** | 0.34 *** | 0.38 *** | — |  |  |  |  |  |
| 6. Distancing | 0.00 | 0.18 ** | 0.30 *** | 0.32 *** | 0.63 *** | — |  |  |  |  |
| 7. Expressive Suppression | 0.19 ** | 0.08 | 0.14 * | 0.28 *** | 0.21 *** | 0.32 *** | — |  |  |  |
| 8. DERS | 0.39 *** | 0.05 | -0.06 | -0.01 | -0.07 | -0.03 | 0.28 *** | — |  |  |
| 9. Age | -0.11 | -0.06 | -0.11 | -0.05 | 0.03 | -0.03 | -0.06 | -0.32 *** | — |  |
| 10. SES | 0.06 | -0.04 | -0.06 | 0.00 | -0.11 | -0.04 | 0.03 | 0.11 * | -0.02 | — |

*Note.* * p < .05, ** p < .01, *** p < .001

Supplemental Table 2. Multiple regression results including distancing moderation terms.

*Predictors B Std Error ß Lower 95% CI Upper 95% CI t p*

| (Intercept)  Situation Selection  Situation Modification  Distraction  Reinterpretation  Distancing  Expressive Suppression  DERS  Age  Gender  SES  Race/Ethnicity  DERS * Distancing  Age * Distancing  Gender * Distancing  SES * Distancing  Race/Ethnicity * Distancing | 6.00 0.52 3.08 1.72 0.92  -7.42  1.14  16.31 -0.11  -3.62  0.35  -12.24  0.55  0.05  -0.19  0.17  3.01 | 24.23  0.75 1.00 1.12 1.28 5.59 1.16 5.84 0.30 8.73 2.54  11.95  1.35 0.07 2.01 0.63  2.71 | 0.07  0.04  0.21 **  0.10 0.06  -0.18  0.07  0.44 ***  0.06  -0.15  0.07 0.01 0.02 0.05  -0.01  0.02  0.18 | -0.10  -0.07  0.07  -0.03  -0.10  -0.38  -0.07  0.30  -0.07  -0.37  -0.06  -0.24  -0.08  -0.08  -0.24  -0.11  -0.14 | 0.24 0.14 0.34 0.23 0.21 0.02 0.21 0.57 0.19 0.06 0.19 0.26 0.13 0.18 0.22 0.15  0.49 | 0.82 0.68 3.06 1.54 0.72  -1.72  0.98 6.41 0.92  -1.41  1.08  0.07  0.41  0.75  -0.09  0.27  1.08 | 0.41 0.50 0.00 0.12 0.47 0.08 0.33 0.00 0.36 0.16 0.29 0.94 0.68 0.45 0.93 0.79  0.27 |
| --- | --- | --- | --- | --- | --- | --- | --- |

Note: * p < .05, ** p < .01, *** p < .001
